# “Something Was Attacking Them and Their Reproductive Organs”: Environmental Reproductive Justice in an Indigenous Tribe in the United States Gulf Coast

**DOI:** 10.3390/ijerph18020666

**Published:** 2021-01-14

**Authors:** Jessica L. Liddell, Sarah G. Kington

**Affiliations:** 1 School of Social Work, Tulane University, New Orleans, LA 70112, USA; 2 Department of Sociology, School of Liberal Arts, Tulane University, New Orleans, LA 70118, USA; skington@tulane.edu

**Keywords:** American Indian, Native American, Indigenous, environmental reproductive justice, reproductive justice, environmental justice, health disparities, qualitative research

## Abstract

Environmental reproductive justice is increasingly being utilized as a framework for exploring how environmental exploitation and pollution contribute to reproductive health and reproductive injustices. However, little research explores how settler colonialism and historical oppression contribute to the physical transformation of land, and how this undermines tribal members’ health. Even less research explores the intersection of environmental justice and reproductive justice among Indigenous groups, especially in the Gulf South, who are especially vulnerable to environmental justice issues due to climate change, land loss, and oil company exploitation, and for tribes that are non-federally recognized. A qualitative description research methodology was used to conduct 31 life-history interviews with women from a Gulf Coast Indigenous tribe. Findings of this study reveal that central components of reproductive justice, including the ability to have children and the ability to raise children in safe and healthy environments, are undermined by environmental justice issues in the community. Among concerns raised by women were high rates of chronic healthcare issues among community members, and issues with infertility. Recognizing Indigenous sovereignty is central to addressing these environmental reproductive justice issues. This research is unique in exploring the topic of environmental reproductive justice among a state-recognized Gulf Coast tribe.

## 1. Introduction

Both environmental justice (EJ), defined as “the fair treatment and meaningful involvement of all people regardless of race, color, national origin, or income, with respect to the development, implementation, and enforcement of environmental laws, regulations, and polices” [1], and reproductive justice (RJ), defined as the right to have children, to not have children, and to parent children in a healthy and safe environment [2], have been documented as issues of injustice among Indigenous communities [3,4,5,6]. The intersection of environmental justice and reproductive justice highlights the ways that environmental injustice leads to reproductive injustice, as described by the emerging lens of environmental reproductive justice (ERJ). Scholars and activists have been increasingly pointing to this as a useful framework to analyze the unique ways in which injustices occur that are related to both the environment and reproduction [7,8], especially among Indigenous populations [3,9]. The documentation of these issues points to the need for increased research and targeted policies to ensure that environmental reproductive justice can be realized among Indigenous communities in the US.

In this article, we use a historically informed approach to explicate the ways that environmental injustice has impacted Indigenous communities in an ongoing, continuous manner beginning at the onset of settler colonialism in the United States. By taking this long historical view, we are able to more clearly demonstrate how the history of settler colonialism, including the genocide and forced removal of Indigenous communities from their land, has led to the injustices experienced by Indigenous communities today, including the contemporary injustices of continued land loss and corporate industrial exploitation of Indigenous land. This connection makes clear the need for policy changes that will get at the root causes of environmental injustices. We then apply the ERJ framework to illuminate how these environmental injustices have led to reproductive injustices among a state-recognized Indigenous tribe in the Gulf South.

### 1.1. Settler Colonialism, Environmental Justice, and Indigenous People

Scholars have noted that settler colonialism should be viewed as an ongoing process, rather than a historical event [10,11]. By taking this approach, we can more clearly see that today’s environmental injustices are not isolated instances but part of the ongoing settler colonial project. Settler colonialism is distinct from colonialism in several important ways which impact environmental injustices experienced by Indigenous groups in the United States. Glenn (2015) notes the importance of the contrasting objectives of classic vs. settler colonialism, and the impact these different goals have on the way settlers engage with Indigenous populations [10]. In classic colonialism, the goal is extraction of the land to send back resources to a different home, often viewing native people as a “resource” (i.e., cheap labor) to exploit. In contrast, the settler colonialist objective is to gain control of and settle the land. In order to do this, settlers first work to eliminate the native people, through methods such as genocide, forced removal and annexation, and assimilation [11]. In the United States, a combination of all three of these have occurred among the Indigenous population at various points throughout history [10,12]. 

To then accomplish the goal of gaining ownership over the land, the settlers usually impose their own political system and version of legality, which is itself a settler colonialist act [13]. This often manifests as the well-known example of coercing Indigenous populations into signing treaties granting land to the settlers [12]. This Western understanding of land ownership tends to vary from the way that Indigenous people view their relationship with the land, where land is often cared for as recognition of the sustenance and value it provides [10]. Similarly, health and well-being for many Indigenous people is viewed holistically. This includes the importance of balance across mental, physical, emotional and spiritual dimensions, and often also necessitates harmony and care of the community and environment [14]. This severing of the relationship with the land then has important implications for the health and well-being of Indigenous populations [9]. This works to force assimilation, potentially continuing the settler colonialist process of elimination of the native population, though Indigenous populations have been particularly resilient in this regard despite these enormous obstacles [15,16]. Hoover et al. (2012) also point out that because Indigenous communities have a stronger reliance on the land, any kind of environmental injustice tends to have greater consequences than would be seen in the general population [3].

The components of settler colonialism inherently give rise to environmental injustices. This began with the initial forced removal of Indigenous tribes from their land, oftentimes being forced onto less desirable settlement areas, and continues with various processes, including the ongoing industrial exploitation of land surrounding Indigenous communities as seen in the tribe investigated here [17,18,19,20]. By using the framing of settler colonialism as structure and an ongoing process, we can see that the environmental injustices (and the corresponding reproductive injustices) experienced by Indigenous populations today are not isolated events, but stem from the onset of settler colonialism, and manifest as part of this ongoing process. Connection to the environment is also a central component of community resilience, and it is undermined by loss and damage to the land [19,20,21]. While this paper investigates the local context specific to the Gulf South United States, it is important to note that environmental injustices among Indigenous people is an unfortunate global trend documented throughout the world, including in Alaska [22,23], Australia [24], and Peru [25].

Environmental injustices are well documented along racial lines in the US, with communities of color being more likely to experience environmental injustices [26,27]. Examples of environmental injustice among Indigenous communities include pollution [28], exploitation from oil and gas companies [29,30,31], and disproportionately bearing the brunt of the effects of climate change (e.g., rising sea levels) [5,6]. Environmental pollution has known deleterious health consequences, leading to significant risks such as shortened life expectancy [32], reproductive health issues [33], and increased rates of infant mortality [34], cancer [35,36] and asthma [37]. Furthermore, this pollution impacts Indigenous food sovereignty, where traditional food practices such as fishing and growing local foods may no longer be considered safe. This can force Indigenous communities to purchase more food, disrupting this relationship with food and their environment, while also leading to a less healthy, more processed diet, linked with the rising rates of diet-related diseases (e.g., diabetes and hypertension) seen among Indigenous populations in the US [38,39]. This is another example of the continuing erasure of Indigenous culture through forced assimilation, compounded by the effects of the globalized industrial food system, demonstrating again the historical continuation of the settler colonial process [40].

### 1.2. Environmental Reproductive Justice

Environmental reproductive justice (ERJ) has emerged as a lens to describe the ways environmental justice and reproductive justice can be looked at simultaneously, rather than as discrete issues [9]. As we have already discussed environmental justice, a brief description of reproductive justice will be useful before we elaborate on the concepts of ERJ.

The reproductive justice framework was developed by women of color activists in response to the mostly white “reproductive rights” movement, which relied heavily on a choice rhetoric, and emphasized access to birth control and abortion. Women of color noted the ways this movement left out the issues that they saw impacting their communities, such as forced sterilization, medical discrimination, and lack of access to a healthy environment [41]. This critical framework, which emphasizes the socio-political-economic factors that impact reproductive autonomy and health, identifies the three core components of reproductive justice described previously. Because the environment can impact both the ability to have children and the ability to raise children in a safe environment, there are inherent overlaps between reproductive justice and environmental justice. Activists and scholars have begun to describe these connections, identifying a distinct environmental reproductive justice framework.

Hoover (2018) describes ERJ as an intersectional analysis of EJ and RJ, noting that it is in their overlap where we can see the way environmental harms impact reproductive justice [9]. Consistent with the RJ and EJ frameworks, ERJ incorporates analysis of how intersectional identities can impact one’s exposure to environmental harms, and reproductive agency [9]. Intersectional theories highlight the ways that multiple identities (for instance race and gender) intersect and interact with each other such that experiences of oppression may be similar or distinct depending on an individual’s social location [42]. Citing Katsi Cook and others in the Akwesasne community as the originators of the term, Hoover (2018) explains that ERJ “includes ensuring that a community’s reproductive capabilities are not inhibited by environmental contamination” [9]. Importantly, in this framework, the negative effects of environmental contamination on both physical and cultural reproduction are highlighted. In addition to expanding the definition of RJ to include ways the environment impacts cultural as well as human reproduction, Hoover (2018) also suggests expanding EJ to more closely make the connections between the environment and reproduction [9]. 

### 1.3. Tribal Context and Background

The tribe interviewed for this project is located in close proximity to the Gulf Coast and includes bayous, wetlands, and river systems, in addition to petroleum infrastructure, such as levees and canals [43,44,45]. The coastal areas are depended upon for both economic and cultural resources, making Indigenous people in this region especially susceptible to climate change [29,43,44,45,46]. Climate change has impacted the area in the form of flooding, exacerbated land loss, multiple hurricanes, and other extreme weather events, which have negatively impacted the health and well-being of tribal members [20,44]. In addition, most tribes located in the Gulf Coast live below sea level, which further increases their risk of continued land loss and flooding [47].

In addition, the tribe has been negatively impacted by oil company infrastructure and exploitation [20,48]. The construction of levees, the dredging of canals, and other oil extraction activities also contribute to land loss and coastal erosion [43]. Events such as the BP oil spill, which was one of the largest petroleum disasters in history, pouring over 4.9 million barrels of oil into the Gulf of Mexico [49], have also caused dramatic and rapid changes which have impacted the environment, health and economic livelihood of tribal members [50]. The impacts of this spill are still being explored, and few studies specifically look at the impact on Indigenous tribes [19,20]. However, research performed with the general population has found that the spill has negatively affected mental and physical health, further exacerbated disparities in healthcare access, and caused social, emotional, and economic stress related to continued under- or unemployment [50,51,52,53].

Climate change and disasters in the Gulf Coast region are increasingly being researched, as are their effects on the impact of climate disasters, such as the impact of hurricanes on those living near the Gulf; however, their impact on Indigenous people has received little attention [44,45,54,55]. This gap is especially troubling because of tribal members’ connection and dependence on the land for cultural, social, and economic resources. In addition, little research has explored the potential impact on tribal members if they are forced to relocate, although tribal members note their concerns that relocation will destabilize individual and tribal identity, and undermine social support and cultural activities [44,45,54,55]. This geographic marginalization also harms the physical well-being and safety of members, since many tribal members reside outside of the levee system, which provides protection from flooding and storm damage [19,29,30]. This has been described as a clear example of “environmental racism” [29].

Relocation is especially sensitive for tribal members since they were forced out of their ancestral homeland during the early 1700s into their current location in the Gulf Coast [47]. However, although there is extensive documentation of their presence in the region, due to political and historical factors, the tribe has been unable to receive federal recognition, although the state has recognized them [19,29,46]. The process for gaining federal recognition is documented as being very political, with guidelines being applied inconsistently, in addition to often being time consuming and requiring extensive economic resources [29,46,47,56].

Tribes which lack federal recognition are unable to receive the benefits and resources afforded to them under federal treaty agreements, which negatively impacts their ability to access resources following disasters [57]. In addition, it undermines the ability of tribal members to manage and control access and use of the environment in their community, including the preservation and handling of sacred locations [20,29,45,46]. The majority of non-federally recognized tribes are located in the southeast region of the U.S. [58].

A lack of federal recognition has negatively impacted the tribe across several dimensions, especially those related to accessing resources and exercising tribal sovereignty. In the United States, tribal sovereignty entails acknowledging Indigenous tribes as independent nations [29]. Constitutional and state laws frequently do not apply to tribal lands and tribal nations have the ability to control many activities which impact and take place on tribal land [29]. Federally recognized tribes are able to enact environmental regulations on their land as they see fit, and this lack of recognition has facilitated oil company exploitation [19,29,30,45]. Without tribal sovereignty, companies have been able to take land that would have otherwise been held in a tribal trust, through targeting individual tribal members, often through using legal documents and agreements that were not always understood by individual members [19,30,45,46]. Not being federally recognized also allowed BP to deny resources to members following the oil spill, since they do not recognize claims made by non-federally recognized tribes [19,29,30,45]. Although environmental justice issues and climate change are important concerns for all individuals, and tribes, not being federally recognized has made this tribe particularly vulnerable to these impacts.

Because tribal members do not reside on a reservation or tribally owned land, members live throughout the coastal region and are not concentrated in one area [29,45]. The fishing and oil industries are the most common employers for tribal members, highlighting the complicated relationship many members have with oil companies [29,45]. Occupational options are in part limited because of a long history of educational discrimination. In addition, because of this history, English is not the first language of all tribal members [16,20,29,45,54]. Although it is becoming less frequent, historically, tribal members would visit Indigenous healers for healthcare [6,45,54]. Environmental shifts may in part be driving this decline, since changes to the land can negatively impact the availability of plants and medicines used in healing [6,45,54]. However, despite the many obstacles faced by tribal members, resilience, self-sufficiency, the importance of family and supporting community members are highly valued [20]. These values have protected tribal members from some of the negative effects of environmental injustice issues, although their lack of federal recognition continues to be an important barrier.

Historically, many tribes located in the Gulf Coast region were both matrilineal and matrilocal societies [59,60,61,62]. Gender roles were also frequently flexible [61,63]. Gender roles changed dramatically following colonization and previously egalitarian and complementary gender roles were replaced by patriarchal values and structures which often negatively affected the role of women [59,61,64]. For example, colonizers would often refuse to do business or negotiate with female tribal members, undermining women’s leadership roles [59,62]. Recent research exploring the roles and expectations of female tribal members in this group has identified that women often take on caregiving roles, act as the center of the family and as a role models and explored recent decolonizing practices in gender relations [60]. However, this same research also found that women are often expected to prioritize their families over their own educational or economic desires and aspirations.

**Figure 1:**
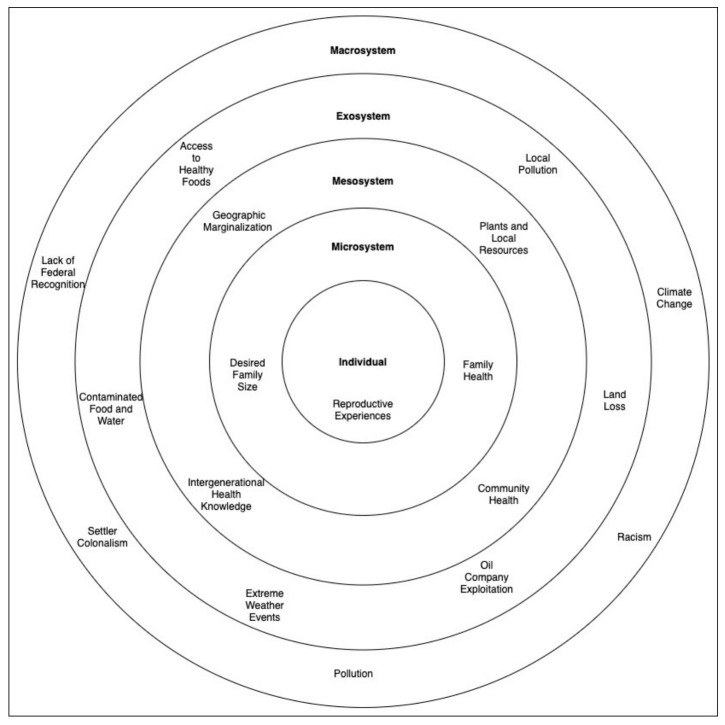
Environmental reproductive justice issues impacting tribal members.

Described previously in this section, Figure 1 illustrates the some of the environmental reproductive justice issues impacting tribal members.

## 2. Purpose

The purpose of this study was to apply the environmental reproductive justice framework to the reproductive health experiences described by women who self-identify as members of a state-recognized Indigenous tribe in the Gulf Coast region of the United States. This study addresses important gaps in the environmental reproductive justice literature, especially in the Gulf Coast region, and for Indigenous groups which are not federally recognized. While violations of both reproductive justice and environmental justice are well documented among Indigenous communities, they are often discussed as discrete issues. By applying the ERJ framework, we can more clearly see the intersection of these issues. The overarching research question addressed is: How do the reproductive health experiences described by women tribal members illustrate the environmental reproductive justice framework?

## 3. Materials and Methods

### 3.1. Research Design

This article utilizes data from a broader research project investigating the reproductive and sexual health experiences of women from an Indigenous tribe in the Gulf South. Best practices to ensure a just and decolonizing research project were employed [16,65,66]. The non-Indigenous lead author was introduced to tribal members through work with another researcher collaborating with the tribe. A community-engaged approach, which is shown to be especially important when working with Indigenous communities [16,65], was utilized at every step of the research process, and the project was designed in collaboration with tribal leaders, who also helped with recruitment and the dissemination of results to the tribe.

A qualitative descriptive methodology was used, with semi-structured in-depth qualitative interviewing as the primary method. Per recommendation from tribal leaders, and in accordance with best practices in conducting research among Indigenous people [16,65,67], the interviews included overtones of a life-history approach. A descriptive methodology was used instead of an overly interpretive methodology to ensure a clear interpretation of interviewees words. This is especially important among Indigenous communities whose well-being has historically been undermined under the guise of research [66,68].

### 3.2. Setting and Participants

Participants included 31 women from a state-recognized Indigenous tribe in the Gulf South of the United States. Participants needed to meet three inclusion criteria: (1) be at least 18 years of age; (2) identify as a woman; and (3) identify as a member of the tribe. Participants self-identified tribal status due to the historical and contemporary barriers to proving tribal identity, which has resulted in discrimination and further marginalization of Indigenous people. The tribe identity remains anonymous due to agreements with the tribal council, and in alignment with best practices in conducting research with Indigenous tribes [16,65].

Ages of participants ranged from 18 to 71, with a median of 51.71. Most participants (81.7%) had a GED or high school degree, with approximately half (51.6%) having additional training or education. Twenty-six participants had children. Participants had an average of 2–3 children and, on average, participants had their first child when they were 20 years old.

### 3.3. Data Collection and Analysis

Institutional Review Board (IRB) approval was obtained from both [blinded for review] University (study #2018-467), and from the tribal council before beginning the study. The study was conducted in accordance with the Declaration of Helsinki. A community-advisory board (CAB) composed of 2 women tribal members helped to develop and pilot the interview questions and assisted throughout the process with recruitment and dissemination of results. The interview guide was designed collaboratively with CAB members to ensure that questions which were most relevant to tribal members were included. CAB members also identified initial seed participants for interviews. Participants were then recruited through purposeful snowball sampling. The first author conducted all interviews and the interviews were conducted in English. Interviews were conducted in a variety of locations, selected according to participants’ preferences. These settings included participants’ homes, community centers, and a local coffee shop, all minimizing travel for participants. Interviews took place between October 2018 and February 2019.

A semi-structured interview guide, informed by the responsive interviewing model [69] was used, with questions following the life trajectory to gain overtones of the life-history approach [70]. The interview guide is included in Appendix A. As is consistent with these approaches, there was ample flexibility in the interviews, allowing for adaptive follow-up questions, and a more natural progression. All participants gave their informed consent for their participant in the study before being interviewed. Interviews were recorded with participant consent, then transcribed by a professional transcription service, and checked for accuracy. Interviews ranged from 30 to 90 min each, with an average length of 66 min. Participants were offered a $30 gift card. Verbatim interview transcripts were analyzed with a conventional qualitative content analysis approach [71] using NVivo software. Data were analyzed inductively, beginning with open coding to identify broad themes, followed by direct coding to identify further subcodes. For example, themes related to environmental issues were first identified by the lead author. Then, through further subcoding and collaborative discussion and analysis of these identified quotes between the first and second author, the final themes described in this manuscript were identified.

Result summaries were shared with all interviewees who consented to being contacted following the study. All participants were given the opportunity to comment on, question, or add to result findings. No participants requested any changes be made to the findings. Research findings were also presented to and approved by the tribal council. When it is safe to hold in-person community events again these results will be further disseminated at additional tribal events. All publications resulting from this research project will also be shared with tribal members. Findings from the greater research project are also informing the potential development of a women’s support group (one of the goals of the project for the CAB members) and tribal health programs.

## 4. Results

Three major themes demonstrating the ERJ framework were identified, namely (a) environmental injustices; (b) community health concerns; and (c) reproductive injustices. While participants were not questioned directly about environmental topics or concerns, almost all women noted environmental changes such as destruction and loss of land, and pollution. Women described their concerns about the impact these environmental impacts were having on their own health, the health of their children, and of the tribal community. We contextualize community health concerns and women tribal members’ reproductive health experiences using the environmental reproductive justice framework.

### 4.1. “If I’s Killing a Bug, What Is It Doing to Me?”: Environmental Injustices

When discussing environmental health and injustice concerns, subthemes mentioned by women included *“Pollution”* and “*Destruction and Loss of Land*”. Participant 31 described seeing pollution in the environment and felt that its effects could be seen in the food chain:

I think, long time ago…cause they didn’t have regulations, when there was the discovery of oil and stuff like that, I believe that has an effect on it. And also…they spray chemicals…they fly up and down the bayou here, with the mosquito trucks, right? So the mosquitoes, all that chemicals is going in the bayou. It’s going on our plants that we eat, or our vegetables. So it’s a cycle.

Participant 16 also described the impact of pollution and spraying for mosquitoes on the environment and health of community members:

We’re like, not putting enough like plant-based stuff around our town, we’re putting more like factories, which is like creating pollution and just like killing our plants…I definitely don’t see, like our earth being like a better one than it is today because of all the pollution. …like the little mosquito thing…. It’s like they blew out stuff to kill mosquitoes. I think that’s very horrible. And it’s like very unnecessary….’Cause then it, and it kind of makes you cough and you’re like, if it’s killing a bug, what is it doing to me?

Participant 12 also discussed the role of air and water pollution on the environment:

That stinking air pollution…Because in the olden days they used to…drink water from the rain and now you can’t do that because of all those planes and everything else…that smell in the water… That smell.

This participant reported a change where she used to be able to drink rainwater growing up but due to increasing industrialization in her community, the water itself began to take on the smell of the air pollution.

Participant 12 also made an explicit connection between the digging of canals by oil companies and the land loss they saw in their community: “If it [the land] keeps having them floods like they’re having there won’t be no more [community name] in many more years…And all that digging they done [for the oil canal].” Participant 21 also attributed much of the pollution and land loss in the environment to oil and gas company presence and exploitation in the region:

So, so I don’t think you’ll hear a whole lot of people say it, but I mean oil and gas exploration around here… Massive amounts of land loss, huge degradation of the environment and people’s health… And although you will never by any ability that would hold up in court, be able to prove it, it’s like we’re also not stupid, right? If you put an open-air dumping pit next to a community and then all of a sudden people in that-this, this is a real example, have elevated levels of lead in their blood, right? It’s like, yeah, cause and effect.

This participant went on to state, however, that many people in the community were resistant to attributing the changes to the environment to oil company exploitation because of their dependence on these companies for employment:

Like a denial of causation basically. So like people live right next to this chemical plant. And [oil company name] puts out…actually puts out this propaganda like…a healthy living calendar. Don’t forget to eat your fruits and vegetables…There’ll be all these beautiful like swamp scenes in the calendar and then they’ll just like drop a little [oil] rig in there, kind of just like make it look like it’s part of the natural [state name] landscape. And so I think that happens a lot here. There’s just a denial of the hazards. There’s a denial of the causation of the hazards, even though they [community members] can literally see it sometimes like they dredge this canal behind your house and that [water] took up half your yard like over the past couple of decades. People will be like, no, that’s hurricanes, hurricanes did that, because that’s the narrative that they [oil companies] push, like oh hurricanes pumping saltwater [causes the land loss].

This participant describes the lengths oil companies go to distract from the impact they are having on the environment, through a propaganda tool of a healthy living calendar. This participant continued to describe the negative impact of the oil industry on the environment:

So does dredging canals [cause saltwater intrusion]. So does like, a well blowing over. Cause they used to drill like next to salt domes and that’s what killed a lot of like the landscape right here. And so I think there’s just like this narrative that gets pushed to protect the industry and they’re very, very good at it…. the irony is not lost on me….It’s just they’ve made us dependent on them. It’s like no other industry really exists around here. So you have to work in oil and gas. Regardless of the cost…Because if that doesn’t, if it’s not here, then you have nothing…And the communities have nothing…that’s just a hard, that’s a hard conversation to have with anyone …So it’s not up to me to be able to say like, you shouldn’t work in this industry because you’re poisoning people in [place name]. You’re eating up the swamps….It’s difficult.

This participant notes the tension between taking action to combat the negative impacts of the oil industry while also acknowledging that working for these companies is a main source of income for tribal members.

### 4.2. “John Wayne Did Not Live long Enough to Kill All of the Indians, so That’s What They Want to Do, Finish Killing the Indians”: Community Health Concerns 

Women described seeing high rates of chronic health diseases in their community and attributed many of these to pollution and toxins in the environment. The experiences described by participants exemplify environmental reproductive justice issues and include violations of two of the core principles of reproductive justice: the right to have children, and the right to raise children in safe and healthy communities. Participant 2 equated modern-day industrial pollution with the colonization of Western expansion and genocide of Indigenous peoples:

You have all of this industrial stuff…We did not have that when we were young. That all came... I think that is why our people have cancer. They were not used to this….This is what will kill... I said, “John Wayne did not live long enough to kill all of the Indians, so that’s what they want to do, finish killing the Indians.

This participant explicitly connected current environmental justice issues with historical genocide of Indigenous peoples, and viewed it as a continuation of settler colonialism. Participant 9 explicitly described the threat facing children because of changes in the environment:

I mean, the children born now are born with more cancerous disease before they even come out of the womb…. I think that’s the major thing…we’re born with the odds stacked against us…I just think that…it’s getting worse…the higher the population, the higher the pollution, the higher…the carbon footprint. You know, the more people, the less land, the less clean water, the less…free space that we have…it’s just kind of taken over.

This participant connects modern industrialization with the increasing cancer deaths seen among Indigenous people. Participant 14 described her experience becoming a community activist after seeing the high rates of health problems affecting women and children in her tribal community, which was located next to an oil-field waste site:

Well, in my community we had an oil-field waste site…I was living in a toxic town…still do….We got together, kids at the bus stop and parents would say their kids were sick with asthma and every month it’s the same thing. And then we realized they had dumped toxic waste inside of our community. So I became an environmentalist, not by choice but….we fought for seven years against the oil and gas company…I was fighting for the rights of my community and my kids to live a normal, happy life as we knew it in a small town….we watched kids with no asthma become asthmatic. We watched kids would grow up with…severe diarrhea, nausea, dizziness, vertigo. It’s different effects from all these chemicals, from the oil and gas industry.

This participant also demonstrates the resilience and strength of female community leaders. As this participant describes, some tribal members resisted the negative impact of oil production and became community activists fighting to protect the health of tribal members. This participant notes that although she did not consider herself an environmentalist previously, she was willing to fight against the powerful oil and gas companies to protect the rights of community and family.

All women spoke about the high rates of cancer they saw among tribal members and in their own families. Most women felt that these rates were increasing and were concerned about what was causing these high rates of cancer. Participant 9 described the disproportionate rates of cancer in her tribal community: “The cancer is ridiculous. Ridiculous. I mean, a while back we had a month where I don’t think we went a single week without having like two people pass away from cancer out of all [tribal name omitted], that were all tribal people. I mean, it was just crazy.” Participant 22 also described seeing high rates of cancer:

Cancer is such on the rise in the Native [community]…I’ve had, 1, 2, so my mom’s…her sister and my grandma…all had cancer, three of the four died. My uncles on my dad’s side, three of the six brothers had cancer…it’s becoming more and more prominent in that area…obviously they should be looking at environment, right…when they’re diagnosing them or looking at their general health and what’s causing it.

Participant 2 also described high rates of cancer and her belief that industrial toxins in the environment have caused this increase:

My husband died of cancer….I have two brothers that died of cancer and then I have my little brother that’s having cancer right now. But I think it’s-environment…And I say that to a lot of people, because I have a few people that had cancer, I think it’s seven…I think it’s coming from the shipyard. Because they sandblast, the sand’s all over. They paint, you have paint going all over….Because cancer, the first one that I ever heard that had a cancer, in my family, was my grandmother, and she was 76-years-old, and that was in 1963. That was the first time that I heard of a family member had cancer.

This participant went on discuss how cancer was viewed and described in her tribal community:

They used to call that [cancer]…It’s a disease that would eat up their flesh, they thought that it would eat up their…The inside of their body, that’s what it was, that’s what they used to call that. They had, they wouldn’t call it a disease, they would say it was a sickness that they had inside their bodies that was eating up inside their bodies. That’s the way they used to-that’s how they used to say it. And then my grandmother, the other [healer] that was there, they said there was nothing they could do, they didn’t know how to treat [heal] for that.

This participant notes that in part because cancer was so uncommon previously, even well-respected healers in the community did not know how to treat it. Participant 3 described seeing high rates of cancer that specifically impacted women, such as breast cancer, in addition to other chronic health conditions such as diabetes, and also connecting it to toxins in the food and environment:

We’re hearing a lot of breast cancer people right now like women….and diabetes. High blood pressure….It could be the food we eat. It’s what I would think. Look in the olden days, they wouldn’t eat no food in no cans and stuff like that. They raised their own stuff and that’s what they would eat.

Participant 22 also described high levels of breast cancer in addition to cervical cancer among tribal women:

Women are getting breast cancer so much earlier now…the few people who I’ve known who have breast cancer…had it before they were 30.… My same little niece, she, um, she has to have surgery next Tuesday for precancerous cells. In her cervix, which is pretty, the doctors said she was fairly young for [it]…she’s 22.

Participant 31 said that the fear of cancer, and the belief that it was being caused by something in the environment, kept one of her relatives from wanting to move back to the region where the tribal community is located:

And you know, like my uncle lives up in north [name of state]. He’s like, I ain’t moving back down. There’s no way we’re moving back down just because of the one reason. Cause everybody in our tribe, I promise you, 75% of the people that die in our tribe is [because of] cancer…. It’s high. So high.

### 4.3. “I Always Had Like Women’s Problems”: Reproductive Injustices

Fertility problems, endometriosis, polycystic ovary syndrome (PCOS), frequent miscarriages, and complications during pregnancy and labor were described by many women. These experiences fall under the reproductive justice category of the right to have children and indicate that many tribal women are currently unable to meet their reproductive goals. Though not all women made an explicit connection between reproductive health issues and the environment, several women reported seeing a direct relationship. Participant 21 described the connection between toxins in the environment and fertility:

I look up a lot of these toxins that are around here. They’re linked to…fertility problems, which is a very prevalent problem around here, which does not seem to be the case a couple of decades ago, like my mom was an only child but my grandmother had a bunch of sisters and like all the generations before them had like tons and tons of kids. There was no problem. And I know more people now that have like tried- try and try and cannot have children.

Participant 29 also described difficulties getting pregnant: “Honestly, it was my body. I couldn’t get pregnant.” Participant 22 described her struggles with infertility and her feelings that PCOS was becoming more common:

It took four years or is it three years [to get pregnant]…I was diagnosed with polycystic ovary syndrome. Which seems to be a trending thing more and more…it’s becoming so much more prevalent without them [women] knowing that they have it.

Participant 17 also described her history of PCOS and other women’s health issues:

I always had like women’s problems, I have PCOS and I have endometriosis, so I always had irregular periods and things like that.” Participant 23 related that she had to get a hysterectomy at a young age, even though she wanted more children, because of excessive bleeding: “I had to have a hysterectomy at 25…I was young…I had a lot of problems with my uterus.”

In addition to difficulties in getting pregnant, carrying a child to term was an obstacle for many women. Participant 9 described her frustration after experiencing multiple miscarriages and not being able to find out what was causing them:

And the third one…I remembered them saying that they would call me, with results to find out maybe what happened, why I keep having these miscarriages and stuff and nobody called.

Participant 3 also discussed having had a miscarriage: “I had a miscarriage. Before she [daughter] was born I lost a baby.

Participant 22 thought that she might have miscarried because of having cancer: “The miscarriage was possibly [because] I did have thyroid cancer.”

For women who did not miscarry, premature birth was another concern identified by tribal members. Participant 14 described giving birth prematurely: “I was…seven weeks early. I had toxemia….So he [son] was a preemie.” Participant 3 also stated that she gave birth prematurely: “So, she [her daughter] weighed two pounds and four ounces…She stayed 42 days in the hospital.” Participant 29 reported having complications during postpartum: “I ended up in the hospital after I had my children, I had postpartum eclampsia.”

Participant 14 described the many reproductive issues she saw in her community, which was located next to an oil-field waste site:

We had two babies born stillbirths…with, cysts on their bodies…And…they were fine [up until their stillbirth]. And then five months into the pregnancy, a lot of times, the baby, [the mothers] wouldn’t feel the baby moving, so they go and do ultrasound and say [the doctors] you’re not going to carry full term, you got to deliver. The babies were affected by some type of abnormal growths on the body, throughout the body…Two spina-bifidas in a small community, which is rare…Just crazy things like that….We had a cluster of women who had total hysterectomy… Usually they would go for a checkup and they had growths in their uterus the size of a cantaloupe or a grapefruit…At a young age…Something was attacking them and their reproductive organs… It still exists and the reason why we had an oil field waste site in our communities is because we were mostly natives.

This participant makes the explicit connection that the reason this waste site was able to be located where it was is because the community was made up of Indigenous people. In these quotes, women describe experiencing a range of reproductive health issues which not only impact their own physical health and well-being, but also their ability to achieve their fertility goals and desires.

## 5. Discussion

These findings highlight the environmental reproductive justice issues experienced by a Gulf Coast Indigenous tribe. Women described both historic and contemporary environmental injustices. They also discussed high rates of chronic diseases and other health issues in the tribal community. Finally, women also noted reproductive health problems for tribal women, including infertility, miscarriage, eclampsia, pre-term birth, and gynecological cancers. In many cases, women made explicit connections between the changes they saw in the environment and their own physical health and that of their community, in addition to the health of the environment itself. It is important to note that there were no interview questions asking about environmental concerns; these issues were all brought up independently by the women tribal members being interviewed, demonstrating the importance of these issues to them. Utilizing the environmental reproductive justice framework, the experiences discussed highlight how the core principals of reproductive justice are not being met in this tribal community due to environmental injustice.

Consistent with previous research, women in this study describe extensive changes to the land, which put them at increased risk for negative health effects because of their close connection to the environment for cultural and economic activities and resources [3,18,19]. Tribal members may also be especially adept at identifying changes in the land because of their close relationship with and time spent in the environment. Increased pollution or other environmental issues can cause individual reproductive issues such as infertility, pre-term birth or eclampsia [72]. In the context of the environmental justice issues described by women, these findings suggest that there may indeed be a connection between these health issues and the changes to the environment.

These findings also highlight the tension between oil companies and the tribal community, since, as one participant notes, tribal members are often dependent on the very infrastructure that is destroying the land for employment. Although, as one participant aptly noted when saying, “[the oil company] made us dependent on them,” the need to be part of the global capitalist economy is itself an effect of settler colonialism. However, forms of resistance and resilience were also reported, and some women ended up becoming activists to combat the impact of oil company exploitation on their communities. Participants also noted their concerns about the safety of the food and water, especially seafood because of oil industry pollution. As eating seafood and spending time on the water are key components of tribal identity, the undermining of their practices acts as a risk factor for both economic, cultural, physical and mental health outcomes [20], as well as a violation of the cultural and social reproduction component of ERJ [9]. Prevention of cultural reproduction also risks further forced assimilation of the tribe, one way settler colonialism can work to eliminate Indigenous people.

Tribal sovereignty and well-being is undermined by their lack of federal recognition, as it has allowed oil companies to profit from the damage to the environment without the industry being held accountable [19,29,30,45]. Tribes that have federal recognition have been able to enforce environmental protections, such as in cases where tribes have used the Clean Air Act to control pollution from companies located outside reservations, or in restrictions placed on water use and pollution for the City of Albuquerque by the Pubelo of Isleta tribe [46,73]. Participants also noted the unlikelihood that they would be able to receive justice for these environmental harms in court, suggesting that they would be likely to have their intelligence challenged if they tried. The failure of the courts to be able to deliver justice to Indigenous tribes in this case harkens all the way back to the early days of settler colonialism, when the settlers’ legal system was imposed to grant Indigenous land to settlers in the first place, as well as to the countless Indigenous treaties broken by the U.S. government [12].

In the face of environmental reproductive injustices, women tribal members demonstrate a strong sense of resiliency. They identified environmental issues as a likely cause of the rise of health issues in the community, as well as the reproductive health issues experienced by themselves and others. Some women have even become environmental activists, working to take on the oil and gas companies themselves. Other participants noted that because tribal participants are dependent on exploitative industries for employment, they are placed in a bind, where they either must deny the risk to the environment caused by these corporations, or deny the causation of the risk, something which has been observed in other communities of color experiencing pollution from the oil industry [74].

## 6. Limitations and Future Research

This study is unique in being the first to use an environmental reproductive justice framework to explore the health experiences of Indigenous women in the south. However, these findings are very contextual and specific to the tribe in this study and may not be generalized to other Indigenous groups. Although women were interviewed across a range of ages to explore generational differences, this study is limited in being cross sectional and using self-report data. It is recommended that future studies use a longitudinal approach to better track environmental and health changes throughout the life of participants. Interviews were also only conducted in English, which, due to the history of educational discrimination experienced by many elder tribal members who do not speak English as their primary language, may have excluded those participants stories and experiences. An additional limitation of this research is that male community members were not interviewed. Future research should explore their viewpoints.

In discussing ERJ, Hoover (2018) describes how many Indigenous people were attracted to the term ERJ because it is unique in exploring how cultural and social reproductive practices are disrupted by environmental injustices, in addition to biological reproduction [9]. Although we noted several examples of this, cultural and social reproduction was not the focus of this article but is an area that warrants further exploration. Future research could also specifically explore women’s beliefs and concerns about breastfeeding as previous research has documented Indigenous women’s worries about contaminants in breastmilk [3].

## 7. Conclusions

This research identifies the environmental reproductive injustice experiences of one Indigenous tribe. This study is unique in applying the environmental reproductive justice framework to a Gulf Coast, and to a non-federally recognized, tribe. It extends previous research looking at the health consequences of environmental changes for Gulf Coast tribes through the environmental justice framework. The results of this research suggest that tribal members are not only exposed to environmental hazards in the form of climate change, pollution, land loss and oil company exploitation, but that these threats act as a continuation of settler colonialism and undermine the health and reproductive capabilities of the tribal community.

A lack of federal recognition has facilitated and exacerbated many of the environmental injustices this tribe has experienced and has barred them from receiving needed resources. In addition to the need for federal recognition, any environmental interventions should meaningfully incorporate the voices of tribal leaders. These findings highlight the urgent need for government and corporations to take action to address and remedy the environmental and health damages that have been caused, in addition to the need for tribal sovereignty over their land. Because of the rapid changes occurring to the environment, and their negative impact on the health of tribal communities, the need for action is urgent.

## Data Availability

The data presented in this study are available on request from the corresponding author. The data are not publicly available due to concerns for tribal privacy.

## Appendix A. Interview Guide

Gender Norms and Beliefs

1. Can you tell me about your family growing up?
2. Who made decisions in your family?
3. Tell me about the relationships in your family.
4. Growing up, were there certain things that only men did? Only women did? Has this changed?

Sexual Health Knowledge and Education

5. Did you have sex education in school?
6. Did your family talk to you about sexual health?
7. When/where did you first learn about sex?
8. How did you first learn about childbirth, reproduction, contraception, sexual health?

Access and Beliefs about Contraception

9. What do people in your community generally think of using birth control?
10. How do women generally get contraception?
11. Do you use contraception?
12. What were your experiences getting/using X?

Beliefs about Sex and Reproductive and Sexual Experiences

13. Can you talk to friends/family about sex?
14. What does reproductive health mean to you?
15. Can you tell me about the first time you needed sexual/reproductive health services?
16. Can you describe your first romantic/sexual experience?

Medical Infrastructure

17. Where is the nearest health facility?
18. Can you tell me about an experience using that facility?
19. What do you think about that facility?

Access to Care

20. Can you tell me about a time you needed sexual/reproductive healthcare?
21. What current programs exist for women’s health?
22. What needs exist?
23. Are there things that make it harder for you to get care?
24. Are you able to see a doctor whenever you need to?
25. How do you usually pay for healthcare?
26. What programs are needed?

Patient–Provider Relationships

27. Can you tell me about your last experience seeing a doctor?
28. Can you talk to your doctor about anything?
29. How do healthcare providers treat tribal members?

Child-Bearing and Rearing Experiences and Resources

30. Do you have children? If no, do you want children?
31. Can you tell me a little bit about being pregnant and giving birth?
32. Can you tell me a little about what it’s been like to raise children?
33. What has been hard/what has been easy?
34. What community supports exist for raising children?
35. Has raising children changed since you were growing up? How?
36. What supports are needed?
37. What are barriers to health, including substance abuse, access to healthy food, financial problems?
38. What else would you like to tell me about your life?

## References

[1] USEPA Environmental Justice. https://www.epa.gov/environmentaljustice.

[2] Price K. (2010). What Is Reproductive Justice? How Women of Color Activists Are Redefining the Pro-Choice Paradigm. Meridians.

[3] Hoover E., Cook K., Plain R., Sanchez K., Vi W., Miller P., Dufault R., Sislin C., Carpenter D.O. (2012). Indigenous Peoples of North America: Environmental Exposures and Reproductive Justice. Environ. Health Perspect..

[4] Singer M. (2018). Climate Change and Social Inequality: The Health and Social Costs of Global Warming.

[5] Tsosie R. (2007). Indigenous People and Environmental Justice: The Impact of Climate Change. Univ. Colo. Law Rev..

[6] Vinyeta K., Whyte K., Lynn K. (2016). Climate Change Through an Intersectional Lens: Gendered Vulnerability and Resilience in Indigenous Communities in the United States.

[7] McCarthy A. (2012). On Fertile Ground: The Environmental and Reproductive Justice Movements as a Unified Force for Reforming Toxic Chemical Regulation. Sustain. Dev. Law Policy.

[8] Willman T. (2012). Toxins in Nail Salons: When Environmental and Reproductive Justice Meet.

[9] Hoover E. (2018). Environmental Reproductive Justice: Intersections in an American Indian Community Impacted by Environmental Contamination. Environ. Sociol..

[10] Glenn E.N. (2015). Settler Colonialism as Structure: A Framework for Comparative Studies of U.S. Race and Gender Formation. Sociol. Race Ethn..

[11] Wolfe P. (1999). Settler Colonialism.

[12] Dunbar-Ortiz R. (2014). An Indigenous Peoples’ History of the United States.

[13] Veracini L. (2010). Settler Colonialism: A Theoretical Overview.

[14] King M., Smith A., Gracey M. (2009). Indigenous Health Part 2: The Underlying Causes of the Health Gap. Lancet Lond. Engl..

[15] Burnette C.E., Figley C.R. (2017). Historical Oppression, Resilience, and Transcendence: Can a Holistic Framework Help Explain Violence Experienced by Indigenous People?. Soc. Work.

[16] McKinley C.E., Figley C.R., Woodward S.M., Liddell J.L., Billiot S., Comby N., Sanders S. (2019). Community-Engaged and Culturally Relevant Research to Develop Behavioral Health Interventions with American Indians and Alaska Natives. Am. Indian Alsk. Native Ment. Health Res. Online.

[17] Liddell J.L., McKinley C.E., Lilly J. Historic and Contemporary Environmental Justice Issues among Native Americans in the Gulf Coast Region of the United States. Stud. Soc. Justice.

[18] Billiot S. (2017). How Do Environmental Changes and Shared Cultural Experiences Impact the Health of Indigenous Peoples in South Louisiana?. Arts Sci. Electron. Theses Diss..

[19] Billiot S., Parfait J. (2019). Reclaiming Land: Adaptation Activities and Global Environmental Change Challenges Within Indigenous Communities.

[20] McKinley C.E., Scarnato J.M., Liddell J., Knipp H., Billiot S. (2019). Hurricanes and Indigenous Families: Understanding Connections with Discrimination, Social Support, and Violence on PTSD. J. Fam. Strengths.

[21] Kirmayer L.J., Sehdev M., Whitley R., Dandeneau S.F., Isaac C. (2009). Community Resilience: Models, Metaphors and Measures. Int. J. Indig. Health.

[22] Ahtuangaruak R. (2015). Broken Promises: The Future of Arctic Development and Elevating the Voices of Those Most Affected by It—Alaska Natives. Polit. Groups Identities.

[23] Sakakibara C. (2009). “No Whale, No Music”: Iñupiaq Drumming and Global Warming. Polar Rec..

[24] Albrecht G. (2016). ‘Solastalgia’: A New Concept in Health and Identity. PAN.

[25] Orta-Martínez M., Finer M. (2010). Oil Frontiers and Indigenous Resistance in the Peruvian Amazon. Ecol. Econ..

[26] Maantay J. (2002). Zoning Law, Health, and Environmental Justice: What’s the Connection?. J. Law. Med. Ethics.

[27] Mohai P., Pellow D., Roberts J.T. (2009). Environmental Justice. Annu. Rev. Environ. Resour..

[28] U.S. EPA (United States Environmental Protection Agency) (2004). Tribal Superfund Program Needs Clear Direction and Actions to Improve Effectiveness.

[29] Crepelle A. (2018). Standing Rock in the Swamp: Oil, the Environment, and the United Houma Nation’s Struggle for Federal Recognition. Belmont Law Rev..

[30] Rhoan E. (2010). The Rightful Position: The BP Oil Spill and Gulf Coast Tribes. San Joaquin Agric. Law Rev..

[31] Sawyer S. (2004). Crude Chronicles: Indigenous Politics, Multinational Oil, and Neoliberalism in Ecuador.

[32] Pope C.A., Ezzati M., Dockery D.W. (2009). Fine-Particulate Air Pollution and Life Expectancy in the United States. N. Engl. J. Med..

[33] Mendola P., Wallace M., Hwang B.S., Liu D., Robledo C., Männistö T., Sundaram R., Sherman S., Ying Q., Grantz K.L. (2016). Preterm Birth and Air Pollution: Critical Windows of Exposure for Women with Asthma. J. Allergy Clin. Immunol..

[34] Chay K.Y., Greenstone M. (2003). The Impact of Air Pollution on Infant Mortality: Evidence from Geographic Variation in Pollution Shocks Induced by a Recession. Q. J. Econ..

[35] Griffith J., Duncan R.C., Riggan W.B., Pellom A.C. (1989). Cancer Mortality in U.S. Counties with Hazardous Waste Sites and Ground Water Pollution. Arch. Environ. Health Int. J..

[36] Vineis P., Husgafvel-Pursiainen K. (2005). Air Pollution and Cancer: Biomarker Studies in Human Populations. Carcinogenesis.

[37] Trasande L., Thurston G.D. (2005). The Role of Air Pollution in Asthma and Other Pediatric Morbidities. J. Allergy Clin. Immunol..

[38] Gracey M., King M. (2009). Indigenous Health Part 1: Determinants and Disease Patterns. Lancet.

[39] McKinley C.E., Ka’apu K., Scarnato J.M., Liddell J. (2020). Cardiovascular Health among U.S. Indigenous Peoples: A Holistic and Sex-Specific Systematic Review. J. Evid. Based Soc. Work.

[40] Coté C. (2016). “Indigenizing” Food Sovereignty. Revitalizing Indigenous Food Practices and Ecological Knowledges in Canada and the United States. Humanities.

[41] Silliman J., Fried M.G., Ross L., Gutierrez E. (2004). Undivided Rights: Women of Color Organizing for Reproductive Justice.

[42] Crenshaw K. (1989). Demarginalizing the Intersection of Race and Sex: A Black Feminist Critique of Antidiscrimination Doctrine, Feminist Theory and Antiracist Politics. Univ. Chic. Leg. Forum.

[43] Austin D.E. (2006). Coastal Exploitation, Land Loss, and Hurricanes: A Recipe for Disaster. Am. Anthropol..

[44] Lambeth T. (2016). Coastal Louisiana: Adaptive Capacity in the Face of Climate Change. Ph.D. Thesis.

[45] Maldonado J.K. (2014). Facing the Rising Tide: Co-Occurring Disasters, Displacement, and Adaptation in Coastal Louisiana’s Tribal Communities. Ph.D. Thesis.

[46] Crepelle A. (2018). The United States First Climate Relocation: Recognition, Relocation, and Indigenous Rights at the Isle de Jean Charles. Belmont Law Rev..

[47] Fitzgerald S.J. (2015). Native Women and Land: Narratives of Dispossession and Resurgence.

[48] Bailey C., Gramling R., Laska S. (2014). Complexities of Resilience: Adaptation and Change within Human Communities of Coastal Louisiana.

[49] Smith L.C., Smith M., Ashcroft P.A. (2011). Analysis of Environmental and Economic Damages from British Petroleum’s Deepwater Horizon Oil Spill. Albany Law Rev..

[50] Fan A.Z., Prescott M.R., Zhao G., Gotway C.A., Galea S. (2015). Individual and Community-Level Determinants of Mental and Physical Health after the Deepwater Horizon Oil Spill: Findings from the Gulf States Population Survey. J. Behav. Health Serv. Res..

[51] Cope M.R., Slack T., Blanchard T.C., Lee M.R. (2013). Does Time Heal All Wounds? Community Attachment, Natural Resource Employment, and Health Impacts in the Wake of the BP Deepwater Horizon Disaster. Soc. Sci. Res..

[52] Croisant S.A., Lin Y.-L., Shearer J.J., Prochaska J., Phillips-Savoy A., Gee J., Jackson D., Panettieri R.A., Howarth M., Sullivan J. (2017). The Gulf Coast Health Alliance: Health Risks Related to the Macondo Spill (GC-HARMS) Study: Self-Reported Health Effects. Int. J. Environ. Res. Public. Health.

[53] Patel M.M., Saltzman L.Y., Ferreira R.J., Lesen A.E. (2018). Resilience: Examining the Impacts of the Deepwater Horizon Oil Spill on the Gulf Coast Vietnamese American Community. Soc. Sci..

[54] Bates D.E. (2016). We Will Always Be Here: Native Peoples on Living and Thriving in the South.

[55] (2016). Simms “Why Would I Live Anyplace Else?”: Resilience, Sense of Place, and Possibilities of Migration in Coastal Louisiana. J. Coast. Res..

[56] Fletcher M.L.M. (2006). Politics, History, and Semantics: The Federal Recognition of Indian Tribes. N. D. Law Rev..

[57] (2004). U.S. Commission on Civil Rights Broken Promises: Evaluating the Native American Health Care System: (510712006-001).

[58] Salazar M. (2016). State Recognition of American Indian Tribes. Natl. Conf. State Legis..

[59] Burnette C.E. (2015). Indigenous Women’s Resilience and Resistance to Historical Oppression: A Case Example From The United States. Affilia.

[60] Liddell J.L., McKinley C.E., Knipp H., Scarnato J.M. (2020). “She’s the Center of My Life, the One That Keeps My Heart Open”: Roles and Expectations of Native American Women. Affilia.

[61] Shoemaker N. (1994). Negotiators of Change: Historical Perspectives on Native American Women.

[62] Weaver H.N. (2009). The Colonial Context of Violence: Reflections on Violence in the Lives of Native American Women. J. Interpers. Violence.

[63] LaFromboise T.D., Heyle A.M., Ozer E.J. (1990). Changing and Diverse Roles of Women in American Indian Cultures. Sex Roles.

[64] Jaimes Guerrero M.A. (2003). “Patriarchal Colonialism” and Indigenism: Implications for Native Feminist Spirituality and Native Womanism. Hypatia.

[65] Burnette C.E., Sanders S., Butcher H.K., Rand J.T. (2014). A Toolkit for Ethical and Culturally Sensitive Research: An Application with Indigenous Communities. Ethics Soc. Welf..

[66] Brown L. (2015). Susan Strega Introduction: From resistance to resurgence. Research as Resistance: Revisiting Critical, Indigenous and Antioppressive Approaches.

[67] Ng A., Fook N. (2007). An Indigenous Curriculum of Place: The United Houma Nations Contentious Relationship with Louisiana’s Educational Institutions.

[68] Denzin N.K., Lincoln Y.S., Smith L.T. (2008). Handbook of Critical and Indigenous Methodologies.

[69] Rubin H., Rubin I. (2005). Qualitative Interviewing (2nd Ed.): The Art of Hearing Data.

[70] John W. (2007). Creswell Five Qualitative Approaches to Inquiry. Qualitative Inquiry and Research Design: Choosing Among Five Approaches.

[71] Milne J., Oberle K. (2005). Enhancing Rigor in Qualitative Description: A Case Study. J. Wound Ostomy Cont. Nurs. Off. Publ. Wound Ostomy Cont. Nurses Soc..

[72] Rosen E.M., Muñoz M.I., McElrath T., Cantonwine D.E., Ferguson K.K. (2018). Environmental Contaminants and Preeclampsia: A Systematic Literature Review. J. Toxicol. Environ. Health B Crit. Rev..

[73] Gilio-Whitaker D. (2019). As Long as Grass Grows: The Indigenous Fight for Environmental Justice, from Colonization to Standing Rock.

[74] Singer M. (2011). Down Cancer Alley: The Lived Experience of Health and Environmental Suffering in Louisiana’s Chemical Corridor. Med. Anthropol. Q..

